# The Influence of *PSCA* Gene Variation on Its Expression and Gastric Adenocarcinoma Susceptibility in the Northwest Chinese Population

**DOI:** 10.3390/ijms160511648

**Published:** 2015-05-21

**Authors:** Wentao Zhang, Ping Liang, Weihua Wang, Peng Dai, Qin Wang, Wei Yan, Jinrong Zhao, Jianbin Sun, Yong Peng, Daxiang Cui, Zhen Yan

**Affiliations:** 1 State Key Laboratory of Cancer Biology, Department of Pharmacogenomics, School of Pharmacy, Fourth Military Medical University, Xi’an 710032, China; E-Mails: zwt1989@fmmu.edu.cn (W.Z.); liangp@fmmu.edu.cn (P.L.); wkwinni@aliyun.com (W.W.); rarfen@163.com (P.D.); maomao_snnu@163.com (Q.W.); zhaojrr@fmmu.edu.cn (J.Z.); sunjianbin815@163.com (J.S.); 2 Department of Pathology, Xijing Hospital, Fourth Military Medical University, Xi’an 710032, China; E-Mail: yan070@sina.com; 3 Department of Radiology, Xijing Hospital, Fourth Military Medical University, Xi’an 710032, China; 4 Institute of Nano Biomedicine and Engineering, Key Laboratory for Thin Film and Microfabrication of Ministry of Education, Research Institute of Translation Medicine, Shanghai Jiao Tong University, Shanghai 200240, China

**Keywords:** prostate stem cell antigen (PSCA), gastric adenocarcinoma, single nucleotide polymorphism (SNP), haplotype, gene expression

## Abstract

Gastric adenocarcinoma (GAC) imposes a considerable health burden around the world. Gene variation in prostate stem cell antigen gene (*PSCA*) has been identified to be associated with GAC risk, while the results showed regional variation. To explore the influence of *PSCA* gene variation on its expression and GAC risk in the Northwest Chinese population, four single nucleotide polymorphisms (SNPs) of *PSCA* were genotyped in 476 GAC cases and 481 controls using MassARRAY system. Two SNPs of rs2294008 (C>T) and rs2976392 (G>A) were identified to be associated with GAC risk. rs2294008, rs2976392 and rs10216533 made up two statistically significant haplotypes (Hap-CGG and Hap-TAG). Additionally, PSCA expression was analyzed by quantitative real time PCR, immunohistochemistry and tissue microarray. The results showed that PSCA expression was decreased in GAC tissues compared with adjacent normal tissues. For normal tissues, PSCA expression was higher with Hap-TA than that with Hap-CG. For GAC tissues, the differentiation degree of Hap-TA was higher than that of Hap-CG. The expression distribution of PSCA in multiple human organs showed disparity. These results suggest that *PSCA* gene variation has a potential effect on its expression and GAC risk in the Northwest Chinese population.

## 1. Introduction

In the world, nearly one million new gastric cancer cases occur each year [1]. Gastric adenocarcinoma (GAC) comprises approximately 90% [2]. The number of cases in developing countries such as China is nearly 3 times as that in developed countries [3], most likely because of the combination of differences in environmental risk factors and ethnicity genetic predisposition [4]. Moreover, as a result of lacking noticeable early symptoms, most GAC patients were not diagnosed until reaching an advanced stage, which often results in low 5-year survival rate [5]. Therefore, there are pressing needs for more specific biomarkers in early warning and diagnosis of GAC [5]. With the development of genotyping technologies, numerous new genetic biomarkers have been identified by association studies of gene variation and cancer susceptibility [6].

Prostate stem cell antigen (PSCA) is a small glycosylphosphatidylinositol (GPI)-anchored cell surface protein belonging to the Thy-1/LY-6 family. It was initially identified and isolated as a tumor antigen over-expressed in prostate cancer tissue [7], and later study showed that PSCA expression increases with high gleason score, advanced stage and bone metastasis in prostate cancer [8]. Although PSCA has been considered to have roles in signal transduction and, indeed, several studies suggest its involvement in cell growth regulation [9,10,11], its precise function remains unknown. A genome wide association study (GWAS) has found gene variation (rs2294008, rs2976392, *etc*.) in *PSCA* to be related to susceptibility of GAC in the Japanese population [12]. However, later findings were not consistent with specific region and population, gastric cancer sites and histological types. Overall, the relationship between *PSCA* gene variation and GAC risk among East Asians was much stronger than that in Caucasians [13]. rs2294008 and rs2976392 may be specifically linked to non-cardia and intestinal subtype GAC respectively [14].

Moreover, GAC is a complex disease, in which one adverse allele of a gene contributes weakly [15]. By haplotype analysis, which has been widely accepted for locus-locus interaction detection [16], we explored the association between *PSCA* gene variation and GAC risk in the Northwest Chinese population, and analyzed its expression in GAC and adjacent normal tissues. At last, the expression distribution of PSCA in multiple human cancer and normal tissues were detected. Our study provided important evidence to reveal the relevance between *PSCA* gene variation and the risk of GAC, which may be helpful to find out new specific biomarkers for the early warning and diagnosis against GAC.

## 2. Results

Based on hapmap database and previous reports, we selected four SNPs from 5'-UTR to 3'-UTR in *PSCA* gene (Table 1). The SNPs were selected with minor allele frequency (MAF) >0.05 in the hapMap Chinese Han population and filtered out those highly linked each other (*D*’ > 0.8). Peripheral blood samples from 476 GAC cases and 481 controls were collected depending on the age and gender requirement (*p* > 0.05, Table 2). All the tested SNPs were in agreement with Hardy-Weinberg Equilibrium (HWE, *p* > 0.05, Table 1).

**Table 1:** Basic information of candidate single nucleotide polymorphisms (SNPs).

SNP ID	Position ^a^	Location	Allele A/B ^b^	HWE-*p*
rs2294008	143,761,931	5'-UTR	T/C	0.618
rs3736001	143,762,807	Exon 2	A/G	0.446
rs2976392	143,762,932	Intron 2	A/G	0.939
rs10216533	143,763,690	3'-UTR	A/G	0.266

^a^ SNP position was obtained from NCBI map viewer; ^b^ A/B stands for minor/major alleles.

**Table 2:** Characteristics of cases and controls.

Variable	Case		Control		*p*
	(*n* = 476)		(*n* = 481)		
Age, mean years (SD)	57.1	(11.5)	56.4	(11.8)	0.334 ^a^
Gender, *n* (%)					
Male	249	(52.3)	247	(51.4)	0.766 ^b^
Female	227	(47.7)	234	(48.6)	

^a^ *p* values were calculated by Student’s *t* test; ^b^ *p* values were calculated by two-sided χ^2^ test.

The results showed that the genotypes of rs2294008 and rs2976392 were associated with GAC risk (Table 3). The allele “T” of rs2294008 was significantly associated with an increased GAC risk (CT + TT *vs.* CC:OR = 1.30, 95% CI (1.01–1.68), *p* = 0.042). The allele “A” of rs2976392 also showed an association with elevated risk of GAC in two models (GA *vs.* GG:OR = 1.37, 95% CI (1.04–1.81), *p* = 0.024; GA + AA *vs.* GG:OR = 1.36, 95% CI (1.04–1.77), *p* = 0.023).

**Table 3:** Genotype frequency of prostate stem cell antigen (*PSCA*) variation and their association with gastric adenocarcinoma (GAC) risk.

SNP ID	Genotype	No. (Frequency)				OR	(95% CI)	*p*
		Case (*n* = 476)		Control (*n* = 481)				
rs2294008	CC	227	(47.8)	261	(54.4)	1.00		
	CT	207	(43.6)	183	(38.1)	1.30	(1.00–1.70)	0.053
	TT	41	(8.6)	36	(7.5)	1.31	(0.81–2.12)	0.272
	CT + TT	248	(52.2)	219	(45.6)	1.30	(1.01–1.68)	0.042 *
rs3736001	GG	382	(80.6)	402	(83.6)	1.00		
	GA	88	(18.6)	74	(15.4)	1.25	(0.89–1.76)	0.195
	AA	4	(0.8)	5	(1.0)	0.84	(0.22–3.16)	0.936
	GA + AA	92	(19.4)	79	(16.4)	1.23	(0.88–1.71)	0.229
rs2976392	GG	190	(43.6)	231	(51.2)	1.00		
	GA	208	(47.7)	184	(40.8)	1.37	(1.04–1.81)	0.024 *
	AA	38	(8.7)	36	(8.0)	1.28	(0.78–2.10)	0.322
	GA + AA	246	(56.4)	220	(48.8)	1.36	(1.04–1.77)	0.023 *
rs10216533	GG	237	(53.6)	256	(54.4)	1.00		
	GA	152	(34.4)	176	(37.4)	0.93	(0.71–1.23)	0.626
	AA	53	(12.0)	39	(8.3)	1.47	(0.94–2.30)	0.093
	GA + AA	205	(46.4)	215	(45.7)	1.03	(0.79–1.34)	0.824

* Statistically significant (*p* < 0.05).

**Figure 1:**
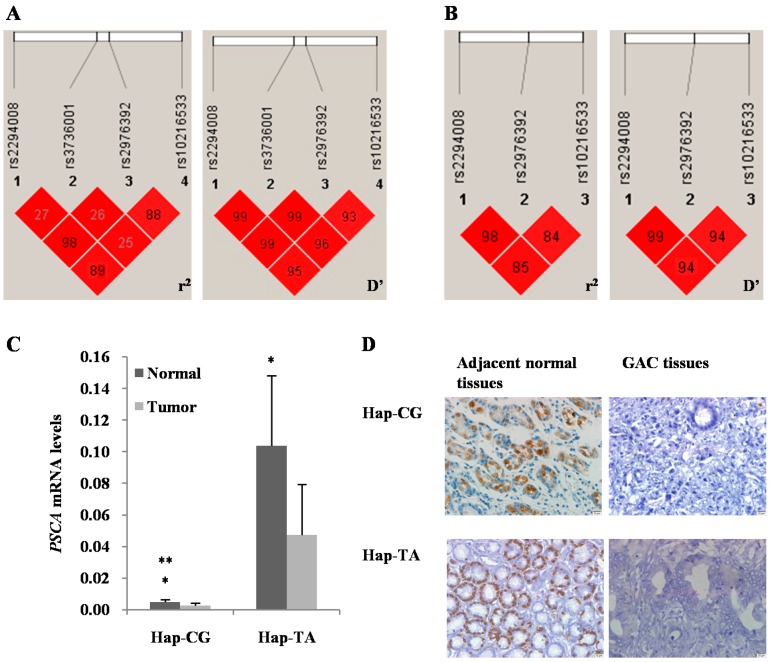
Linkage disequilibrium (LD) and PSCA expression analysis. (**A**) LD of four SNPs in *PSCA* gene (rs2294008, rs3736001, rs2976392 and rs10216533); (**B**) LD of three SNPs in *PSCA* gene (rs2294008, rs2976392 and rs10216533). Values in squares were the pair-wise calculation of *r*^2^ (**left**) and *D*’ (**right**); (**C**) qRT-PCR results showed a significant decrease of *PSCA* mRNA levels in GAC tissues compared with their adjacent normal tissues (Normal *vs.* tumor: Hap-CG, 1.90-fold, *p* = 0.028; Hap-TA, 2.19-fold, *p* = 0.029). *PSCA* mRNA was higher with Hap-TA than that with Hap-CG in normal tissues (Hap-TA *vs.* Hap-CG: 20-fold, *p* = 0.042). Results were expressed as the ratio of the CT value of the *PSCA* gene transcript to that of β-actin. The mean ± standard error (SE) was shown. * *p* < 0.05, normal *vs.* tumor; ** *p* < 0.05, Hap-TA *vs.* Hap-CG; (**D**) Immunohistochemistry (IHC) results revealed that PSCA protein was expressed in differentiated gastric epithelial cells, but silencing in most of GAC tissues (*p* < 0.01). For normal tissues, PSCA expression was higher with Hap-TA than that with Hap-CG (*p* < 0.05). For GAC tissues, the differentiation degree of Hap-TA was higher than that of Hap-CG (*p* < 0.05), Scale bar = 20 μm, Magnification: ×400.

Then the linkage disequilibrium (LD) and haplotype analysis of these four SNPs were conducted (Figure 1A,B and Table 4). A relatively stronger LD was detected between rs2294008 and rs2976392 (*r*^2^ and *D*’ > 0.9, Figure 1A). The Hap-CG accounted for 67% of GAC cases was related to a decreased GAC risk, and Hap-TA was associated with an increased GAC risk with a frequency of 32% in GAC cases, although they did not reach the statistical significance (Hap-CG: OR = 0.83, 95% CI (0.67–1.01), *p* = 0.065; Hap-TA: OR = 1.21, 95% CI (0.99–1.48), *p* = 0.065. Table 4). Additionally, we found that rs10216533 was linked with rs2294008 and rs2976392 (*r*^2^ > 0.8 and *D*’ > 0.9, Figure 1B), which formed three main haplotypes (Table 4). Among them, Hap-CGG accounted for 64.3% and showed a protective effect (OR = 0.782, 95% CI (0.64–0.96), *p* = 0.020) in GAC cases; however, a 4% Hap-TAG exhibited a 12.283 times high risk (OR = 12.283, 95% CI (3.75–40.27), *p* < 0.001) in GAC cases.

According to above results, two haplotypes with higher frequencies, Hap-CG and Hap-TA, showed stronger potential relationships to GAC risk. Next, we analyzed the expression of PSCA by the two haplotypes in GAC and adjacent normal tissues. Quantitative real time PCR (qRT-PCR) showed that the mRNA levels of *PSCA* in GAC tissues were decreased compared to their adjacent normal tissues in the two haplotypes (Normal *vs.* tumor: Hap-CG, 1.90-fold, *p* = 0.028; Hap-TA, 2.19-fold, *p* = 0.029. Figure 1C). In addition, the mRNA level of Hap-TA was higher than that of Hap-CG in normal tissues (Hap-TA *vs.* Hap-CG: 20-fold, *p* = 0.042; Figure 1C). Then the protein expression of PSCA was measured by IHC assay. The results showed that PSCA protein expressed in differentiated gastric epithelial cells, but was silencing in most of GAC tissues (*p* < 0.01, Figure 1D). In normal gastric tissues, the PSCA protein expression with Hap-CG was lower than that with Hap-TA (*p* < 0.05, Figure 1D). Furthermore, the Hap-CG was associated with poor differentiation, and the Hap-TA was related to well differentiation (*p* < 0.05, Figure 1D).

**Table 4:** Haplotype frequency of *PSCA* variation and the association with GAC risk.

Haplotype ID	Frequency ^a^		OR (95% CI)		Fisher’s *p*	Pearson’s *p*	SNP No. and Haplotype
	Case	Control					
							**1–3**
1	0.67	0.72	0.83	(0.67–1.01)	0.065	0.065	C–G
2	0.32	0.28	1.21	(0.99–1.48)	0.065	0.065	T–A
							**1**–**3**–**4**
3	0.64	0.71	0.78	(0.64–0.96)	0.020 *	0.020 *	C–G–G
4	0.29	0.28	1.08	(0.87–1.33)	0.478	0.478	T–A–A
5	0.04	0.00	12.28	(3.75–40.27)	0.00 *	0.00 *	T–A–G

^a^ Only haplotypes with frequencies of ≥3% are shown; * Statistically significant (*p* < 0.05).

**Figure 2:**
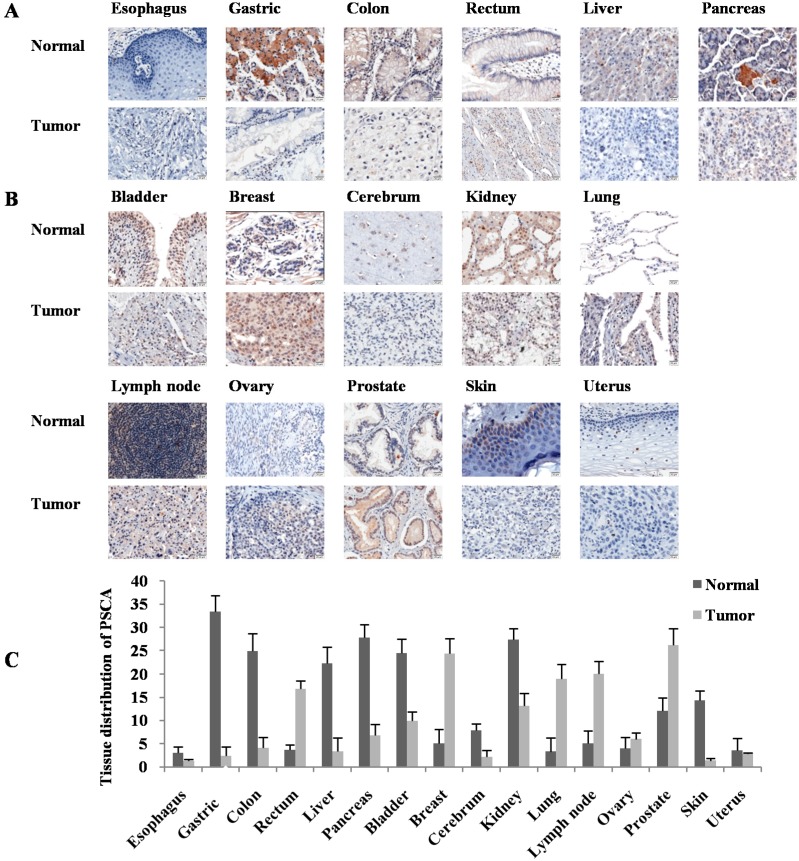
Analysis of PSCA protein expression and distribution in human normal and tumor tissues by tissue microarray. (**A**) Digestive system. Esophagus, negative in both normal and tumor tissues; gastric, colon, liver and pancreas: Positive in normal, negative or down-regulated in tumor tissues; rectum: Negative in normal, and positive in tumor tissues, Scale bar = 20 μm, Magnification: ×200; (**B**) Non-digestive system. Bladder, cerebrum, kidney and skin: Positive in normal, and negative in tumor tissues; breast, lung, lymph node, ovary and prostate: Negative in normal, and positive in tumor tissues; uterus: Negative expression in both normal and tumor tissues, Scale bar = 20 μm, Magnification: ×200; (**C**) Semiquantitative comparison of PSCA immunostaining scores between normal and tumor tissues by IHC.

To examine the PSCA expression in multiple human cancer and normal tissues, a tissue microarray was stained with anti-PSCA antibody. Six digestive system organs were selected, including esophagus, gastric, colon, rectum, liver and pancreas (Figure 2A). The results showed that PSCA expression was positive in the cytoplasm of fundic gland cells in gastric tissue. However, it was not found in mucosal gland epithelial and GAC tissues, which was consistent with our findings above. Here, we also indicated that PSCA was expressed in normal tissues of colon, liver and pancreas, decreased or silenced in their cancer tissues. In the rectum, in contrast, PSCA was negative in normal tissues, but positive in the cancer tissues. Moreover, the expression of PSCA in esophagus was negative in both normal and cancer tissues. The disparities were also existed in other tissues (Figure 2B). PSCA expression was positive in normal tissues of bladder, cerebrum, kidney and skin, and negative in their corresponding cancer tissues. However, the staining was positive in cancer tissues of breast, lung, lymph node, ovary and prostate with a negative staining for normal tissues respectively. In addition, PSCA expression was not detected in both normal and cancer tissues of the uterus.

## 3. Discussion

In the present study, we confirmed that the genotypes of rs2294008 and rs2976392 were associated with GAC risk. Moreover, strong LD and five major haplotypes were observed in the block. It is important to note that SNPs rs2294008 and rs2976392 were in a relatively stronger LD and more responsible for GAC risk according to the odd ratios they predicted in haplotype analysis. On the other hand, however, only the global haplotypes, added rs10216533 together with rs2294008 and rs2976392 into consideration, were statistically significant. Previous study identified that rs10216533 was associated with risk of estrogen receptor negative breast cancer in Korean females [17]. However, its relationship with GAC risk remains unknown. We speculate that rs10216533 may also play important roles in GAC carcinogenesis, which needs to be verified in a larger sample study.

Considering the proportion and statistical power of each haplotype, we analyzed the expression of PSCA in GAC and adjacent normal tissues by haplotypes made up by rs2294008 and rs2976392 (Hap-CG and Hap-TA). We observed a relatively lower level of PSCA expression in GAC tissues compared with their adjacent normal tissues by qRT-PCR, IHC and tissue microarray. Here, the results revealed that Hap-CG was accompanied by lower PSCA expression, while Hap-TA accompanied by a higher level. Moreover, an association was identified between *PSCA* haplotype and GAC differentiation. In the literature, a GWAS revealed that *PSCA* gene variation confers susceptibility to urinary bladder cancer [18], later studies confirmed the association in specific population and analyzed the gene expression by genotypes of rs2294008 [19,20]. Wang’s research found that normal tissues adjacent to tumors with T allele had relatively weak expression of *PSCA* mRNA than those with C allele in the Chinese population, while Fu’s results showed that T risk allele of rs2294008 was associated with increased *PSCA* mRNA expression in bladder tumor samples from a population with European descent. This variation may be due to the differences of region and ethnic. In our population, we identified that specific *PSCA* haplotype has potential effects on gene expression, which might participate in the GAC progression and development. To our knowledge, this is the first original study to comprehensively evaluate the relevance of *PSCA* haplotype on gene expression and GAC susceptibility among Northwest Chinese individuals.

It was interesting that, among the four SNPs selected in the present study, no matter genetic model or LD and haplotype analysis, only three SNPs (rs10216533, rs2294008 and rs2976392) in non-coding regions of the *PSCA* gene were observed to be associated with GAC susceptibility. However, rs3736001, located in exon 2 of *PSCA*, causing a Glu to Lys mutation at protein level, showed no significant association with GAC risk. The three SNPs in non-coding regions, rs2294008, rs2976392, and rs10216533, were located in 5'-UTR, intron and 3'-UTR respectively, which are widely accepted regulation region of a gene [21,22,23]. So we speculate that the variation in non-coding regions of *PSCA* that modulate the gene expression in turn contributes to the GAC susceptibility.

Previous studies demonstrated that gene variation in *PSCA* is associated with the risk of many diseases, such as gastric cancer [12], esophageal squamous cell carcinoma [24], duodenal ulcer [25], prostate cancer [26], urinary bladder cancer [18,19,20], breast cancer [17], *etc.* However, the expression distribution of PSCA in different normal and cancer tissues showed diversity [27], and there is still no tissue distribution of PSCA in specific population published until now. By multiple human organ tissue microarray, we revealed a differential expression of PSCA in human organs, which reminded us that PSCA might have complex functions in the carcinogenesis of different cancers.

In conclusion, our study provided new evidence regarding the effect of *PSCA* gene variation on its expression, which may be involved in the susceptibility and progression of GAC in the Northwest Chinese population.

## 4. Experimental Section

### 4.1. Patients and Samples

The peripheral blood samples were collected from 476 patients with GAC between 2009 and 2011. The control blood samples were from 481 volunteer individuals without known malignancies in 2011. All subjects were collected at the Xijing Hospital of the Fourth Military Medical University (FMMU) in Xi’an city, China. GAC cases were recently diagnosed and histologically confirmed. All of the chosen subjects were Han Chinese living in Xi’an city and its surrounding areas. Informed consent was given by all the subjects for participation in this study according to the ethical rule of the Fourth Military Medical University.

### 4.2. SNP Selection and Genotyping

Four candidate SNPs located in the *PSCA* gene were selected and systematically screened. All the SNPs have a MAF > 0.05 in the HapMap Chinese Han population and filtered out those highly linked each other (*D*’ > 0.8).

Genomic DNA was extracted from the peripheral blood using a Blood DNA Extraction Kit (TIANGEN, Beijing, China). DNA concentration was measured by nanodrop2000 (Thermo Fisher Scientific, Waltham, MA, USA). The isolated genomic DNA was stored at −20 °C. MassARRAY system (Sequenom, San Diego, CA, USA) was used for genotyping. The data management was conducted by Sequenom Typer 4.0 Software (Sequenom, San Diego, CA, USA).

### 4.3. Quantitative Real-Time PCR

E.Z.N.A.TM FFPE RNA Kit (Omega, Norcross, GA, USA) was used to isolate total RNA from paraffin embedded tissues. The required RNA was then subjected to reverse transcription to prepare cDNA, which was applied for qRT-PCR using the PrimeScript™ RT Master Mix (Takara, Tokyo, Japan). The primers of *PSCA* were: forward 5'-CCACCCTTAACCCTGTGTTC-3' and reverse 5'-AAACTCCCAGGAACTCACGTC-3'. Relative gene expression was analyzed by a 7500 fast real-time PCR system (Applied Biosystems, Foster City, CA, USA). Human β-actin was used as an endogenous control. For each sample, the difference in threshold cycles for each *PSCA* copy was calculated by 2^−Δ*C*t^.

### 4.4. Immunohistochemistry and Tissue Microarray

Paraffin-embedded tissue specimens were deparaffinized in turn with xylene, ethanol and PBS. Antigen retrieval was carried out in 100 mM sodium citrate buffer in 100 °C for 20 min. Subsequently slides were immersed in 3% hydrogen peroxide in methanol for 15 min to block endogenous peroxidase activity. Nonspecific binding was blocked with 5% normal goat serum over night at 4 °C. The sections were incubated for 2 h at room temperature with the mouse anti-PSCA antibody (Abcam, ab56338, 1:250 dilution), and then with HRP conjugated anti-mouse IgG antibody. The staining was examined by at least three experienced pathologists.

### 4.5. Statistical Analysis

Statistical analysis was undertaken using Microsoft Excel and SPSS 16.0 statistical package (SPSS, Chicago, IL, USA). All *p* values in this study were two-sided. A *p* ≤ 0.05 was considered the threshold of statistical significance. Genotype frequencies in control subjects for each SNP were tested for departure from HWE using an exact test. Genotype frequencies of case and control subjects were compared using the χ^2^ test [28]. Odds ratios (OR) and 95% confidence intervals (CI) were calculated by unconditional logistic regression analysis [29]. We used the Haploview program to estimate the pair wise linkage disequilibrium between markers and partition haplotype blocks.
